# Giant Power Output from Ionic/electronic Hybrid Nanocomposite Thermoelectric Converter Under Constant Temperature Gradient

**DOI:** 10.1002/advs.202406589

**Published:** 2024-11-23

**Authors:** Siqi Liu, Mingxia Zhang, Junhua Kong, Hui Li, Chaobin He

**Affiliations:** ^1^ Department of Materials Science & Engineering National University of Singapore 9 Engineering Drive 1 Singapore 117574 Singapore; ^2^ Institute of Materials Research and Engineering Agency for Science, Technology and Research, (A*STAR) Singapore 138634 Singapore; ^3^ Hubei Key Laboratory of Plasma Chemistry and Advanced Materials Hubei Engineering Technology Research Center of Optoelectronic and New Energy Materials School of Materials Science and Engineering Wuhan Institute of Technology Wuhan 430205 China

**Keywords:** carbon nanotube, electrospinning, heat harvesting, ionogel, thermoelectric converter

## Abstract

Thermoelectric (TE) materials that directly convert heat to electricity are of great significance for sustainable development. However, TE generators (TEGs) made from electronic TE materials suffer from low Seebeck coefficient (10^−2^–10^0^ mV K^−1^). While ionic TE capacitors based on ionic conductors exhibit high thermovoltage (10^0^–10^2^ mV K^−1^), ionic TE capacitors provide power discontinuously only under variation of temperature gradient as ions cannot transport across electrodes to external circuits. Herein, an ionic/electronic hybrid nanocomposite TE converter (NCTEC) by integrating carbon nanotube/polylactic acid nanofibrous fabrics (CPNF) with gelatin ionogel is reported. The resulting NCTEC exhibits a record‐high output power density normalized by squared temperature gradient (*P_ave_/ΔT^2^*) of 1.72 mW m^−2^ K^−2^ and realizes continuous power output (over 12 h) at a constant temperature gradient, which is among the highest reported power output for TE converters and can be attributed to the combination of substantial increase in interfacial capacitive effect between ionogel and CPNF and an optimized electrical property of the CPNF. The work provides an effective strategy to overcome the limitations of both TEGs and ionic TE capacitors.

## Graphical abstract

A novel ionic/electronic hybrid nanocomposite TE converter (NCTEC) by integrating carbon nanotube/polylactic acid nanofibrous fabrics (CPNF) with gelatin ionogel is developed. A record‐high output power density is realized by NCTEC with continuous power output at a constant temperature gradient, which is contributed by substantially increased interfacial capacitive effect at interpenetrating ionogel/CPNF interface and an optimized electrical property of CPNF.

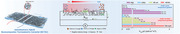

## 1 Introduction

Among the collectible energy sources, heat has been one of the main sources of energy in life and production. Due to the low efficiency of heat utilization, over 60% of energy is dissipated in the environment in the form of waste heat, where low‐grade waste heat (<500 K) accounts for around two‐thirds of the total.^[^ ^1^ ^]^ Moreover, energy harvesting strategies to steadily power distributed wearable electronic devices from the body or surroundings have attracted huge attention recently.^[^ ^2^ ^]^ As such, thermoelectric (TE) materials that convert heat energy such as waste heat or body heat directly to electricity are important for sustainable development.^[^ ^3^ ^]^ Conventional TE materials are based on the Seebeck effect arising from the charge carrier accumulation under the temperature gradient of electronic materials such as inorganic or organic semiconductors. The TE performance of these electronic TE (e‐TE) materials is evaluated by the figure of merit (ZT), *ZT*  = *S* ^2^ σ*T*/κ, where *S* is the Seebeck coefficient, *σ* is the electrical conductivity, *T* is the absolute temperature and *κ* is the thermal conductivity.^[^ ^4^ ^]^ However, the challenge for developing high‐performance e‐TE materials is their small Seebeck coefficient which is usually in the range of 10^−2^–10^0^ mV K^−1^ and the ZT value is mostly below 1 at room temperature.^[^ ^5^ ^]^

On the other hand, ionic TE (i‐TE) materials including ionic conductors such as ionogels,^[^ ^6^ ^]^ ion‐conductive hydrogels^[^ ^7^ ^]^ and polyelectrolytes^[^ ^8^ ^]^ exhibit high thermovoltage of 10^0^–10^2^ mV K^−1^ arising from Soret effect,^[^ ^9^ ^]^ which are two to four orders magnitude higher than that of e‐TE materials.^[^ ^10^ ^]^ The ionic TE performance can be evaluated with the ionic figure of merit (ZT_i_), $ZT_{i}=S_{i}^{2}\;\sigma_{i}T/\kappa$, with *S_i_* and *σ_i_* as the ionic Seebeck coefficient and ionic conductivity, respectively.^[^ ^11^ ^]^ It has been reported that ZT_i_ values of i‐TE materials even up to 1.5–6.1 at room temperature could be achieved by various enhancement methods.^[^ ^6^, ^8^, ^12^ ^]^ However, unlike the TE generators made from e‐TE materials, ions in i‐TE materials cannot transport across the electrodes to the external circuit and it alone cannot generate electricity under static temperature gradient, but rather supply power only during the variation of the temperature gradient in ionic TE capacitors, which is an uncommon form of waste heat.^[^ ^13^ ^]^ The working modes of most ionic TE capacitors reported in the literature are divided into four stages with temperature fluctuation and connect/disconnect to the external load alternatively. Besides, only two stages can actually do work, which largely limits the TE output efficiency.^[^ ^10^, ^13^, ^14^ ^]^

To address the problems of low TE voltage and efficiency of e‐TE materials and discontinuous working modes of i‐TE material, TE converters (TECs) that integrated e‐TE and i‐TE materials with distinct device structures were reported, including hybrid ionic/electronic (i/e) TECs assembled with two separated i‐TE and e‐TE layers^[^ ^15^ ^]^ and mixed i/e TECs constructed with homogenously mixed e‐TE and i‐TE materials.^[^ ^16^ ^]^ Both types of TECs enabled electricity harvesting from temperature gradients like TE generators (TEGs) and temperature fluctuation like ionic TE capacitors. However, because of the separated i‐TE and e‐TE layers of hybrid i/e TECs, the interface contact area in between is quite small with limited interface capacitance. Therefore. The thermovoltage enhancement and power output performance by i‐TE layer integration of hybrid i/e TECs is not so significant only 2–3 times compared to TEGs made from neat e‐TE materials. In contrast, mixed i/e TECs exhibit a much higher transient thermovoltage to mV range that is comparable to i‐TE material, but only when the e‐TE materials loading is low (<2%). Thus, mixed i/e TECs typically exhibit both low electrical and ionic conductivity that hinders the power output performance. Therefore, the TE performance of the reported i/e TECs is still poor and the output power is in the pW range under temperature fluctuation profiles with specific triangle thermal cycles.^[^ ^15^, ^16^ ^]^

Herein, we report a high‐performance i/e hybrid nanocomposite thermoelectric converter (NCTEC) by integrating carbon nanotube/polylactic acid nanofibrous fabrics (CPNF) and gelatin ionogel, which realized remarkably enhanced power output under steady temperature gradient. The NCTEC is of novel design with partially interpenetrating structure of e‐TE material and i‐TE material at the interface in between. The formation of the unique i/e interface structure was attributed to the nanofibrous structures and rough surface of electrospun CPNF, where gelatin ionogel can partially infiltrate into CPNF and create significantly increased surface area between the i/e interface, boosting the interface capacitive effect, thereby enhancing the output power. Besides, the resistance of the e‐TE CPNF was optimized to obtain a higher output performance and realize the continuous power output of the NCTEC under a steady temperature gradient. As a result, a record‐high output power density normalized by the squared temperature gradient (*P_ave_/ΔT^2^*) of 1.72 mW m^−2^ K^−2^ and a 2‐h energy density (*E_2h_*) of 12.4 J m^−2^ with only 1 K of the steady temperature gradient. Moreover, our NCTEC shows the capability of continuous power generation to operate continuously over 12 h and for several consecutive days under operate‐rest‐operate working mode. Our work provides an effective route to boost the power output and realize the continuous power generation of hybrid i/e TE converters under a constant temperature gradient, which is expected to advance this novel strategy toward practical use.

## 2 Results and Discussion

### 2.1 Device Architecture and Morphology of NCTEC

**Figure 1:**
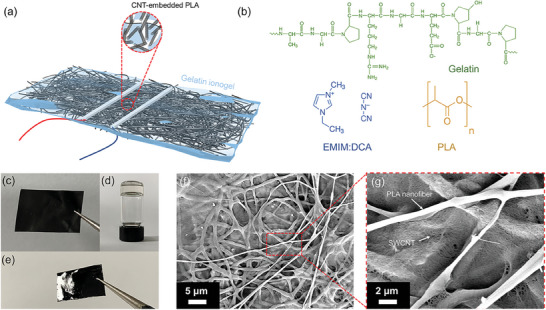
a) Schematic illustration of as‐prepared NCTEC with CNT‐embedded PLA nanofibrous composite fabrics and gelation ionogel; b) Chemical structures of gelatin, EMIM:DCA and PLA; Digital photo of c) a piece of CPNF, d) gelatin ionogel and e) gelatin ionogel uniformly coated on the CPNF; f,g) SEM images of CPNF showing CNT embedded among PLA nanofibers.

An NCTEC was designed and fabricated by applying gelatin/1‐ethyl‐3‐methylimidazolium dicyanamide (EMIM:DCA) ionogel onto carbon nantube (CNT)‐embedded polylactic acid PLA nanofibrous composite fabrics as demonstrated in **Figure** **1**a and the detailed fabrication procedures were explained in Note S1 (Supporting Information). The chemical structures of gelatin, EMIM:DCA, and PLA are shown in Figure 1b. The free‐standing CPNF was fabricated through the innovative electrospray‐on‐electrospinning technique, as illustrated in Figure 1c.^[^ ^17^ ^]^ The gelatin aqueous solution was able to form ionogel upon the addition of EMIM:DCA (Figure 1d) and the gelatin/EMIM:DCA aqueous solution can be uniformly coated on the surface of CPNF (Figure 1e). As shown in surface and cross‐sectional scanning electron microscopy (SEM) images in Figure 1f,g and Figure S1a (Supporting Information), the electrospray CNT was embedded into the simultaneously electrospun PLA nanofibers and formed interpenetrating networks in CPNF. Typically, the electrospun nanofibrous membranes exhibit porous structures with high specific surface area and high porosity.^[^ ^18^ ^]^ SEM characterization of neat PLA nanofibrous membrane confirmed that micron‐sized pores were formed by the PLA nanofibers as exhibited in Figure S2 (Supporting Information). Consequently, a rough surface and high surface area of CPNF were expected due to the CNT embedded in the porous PLA electrospun fabrics.^[^ ^19^ ^]^ Further, SEM observation of the cross‐sectional interface of CPNF and NCTEC proved that the gelatin ionogel infiltrated through part of CPNF and formed i/e interpenetrating region at the interface (Figure S1, Supporting Information).

### 2.2 TE Performance of NCTEC

**Figure 2:**
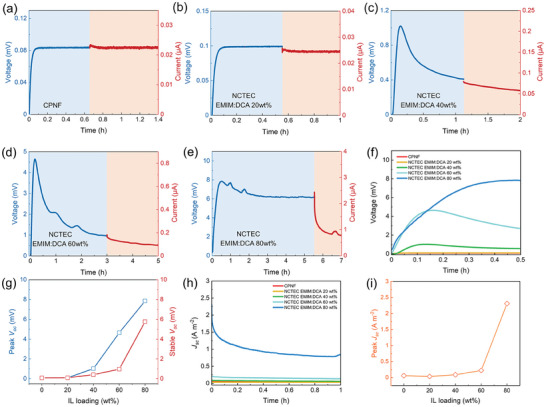
Open circuit thermovoltage (*V_oc_*) and short circuit current (*I_sc_*) profiles with 1 K of temperature gradient (*ΔT*) applied of a) CPNF, and NCTECs consisted of CPNF and gelatin ionogels with IL loadings in gelatin ionogels of b) 20 wt.%, c) 40 wt.%, d) 60 wt.% and e) 80 wt.%; The comparison f) *V_oc_* profile, g) peak *V_oc_*, stable *V_oc_* values, h) *J_sc_* profile and i) peak *J_sc_* of CPNF and NCTECs with different ILs loading in gelatin ionogels. (All samples were tested with a heating rate of 0.7 K min^−1^).

Prior to the testing of NCTEC, the TE properties of the CPNF and gelatin ionogels were measured. The gelatin ionogel with 80 wt% EMIM:DCA loading showed a *σ_i_* of 0.60 S m^−1^ and *S_i_* of 21.9 mV K^−1^ at room temperature at relative humidity of 80% (Figures S3, S4 and Table S1, Supporting Information) which is consistent with the reported values in literature.^[^ ^12^, ^16^ ^]^ The CPNF exhibited a *σ* of 47.6 S m^−1^ *S* of 81.8 µV K^−1^ (Table S1 and Figure S5, Supporting Information). **Figure** **2**a–e showed the open‐circuit voltage (*V_oc_*) and short‐circuit current (*I_sc_*) of the samples applied with temperature gradient (*ΔT*) of 1 K (Temperature profile shown in Figure S6, Supporting Information). Without the integration of gelatin ionogel, the CPNF showed steady thermovoltage under a constant temperature gradient (Figure 2a). As indicated in Figure 2b–e, the thermovoltage profile is extremely sensitive to the EMIM:DCA loadings in gelatin ionogel as it significantly affects the ionic thermoelectric properties of the gelatin ionogel.^[^ ^12e^ ^]^ The NCTEC coated with gelatin ionogel of 20 wt.% EMIM:DCA loading didn't change its e‐TE behavior with steady thermovoltage measured. Further increase in EMIM:DCA loading of coated gelatin ionogel to 80 wt.% led to significantly increased thermovoltage and appearance of transient voltage peak of the NCTECs (Figure 2f,g), where the highest peak *V_oc_* of 7.9 mV can be obtained at 80 wt.% EMIM:DCA loading after heating started. This significant increase in thermovoltage of NCTEC is mainly attributed to increased ionic conductance of the gelatin ionogel with higher loading of EMIM:DCA and the large ionic thermopower of gelatin ionogel with the presence of EMIM:DCA where cations migrated to the cold end as a result of Soret effect. The *V_oc_* profiles of the NCTECs then decay over time toward stabilized voltage values which were much higher than the *V_oc_* of CPNF. This phenomenon is mainly ascribed to the capacitive effect between gelatin ionogel with cation accumulation and CPNF with hole accumulation.^[^ ^15^, ^16^, ^20^ ^]^ Therefore, the negative charges in CPNF tend to migrate to the i/e interface to balance the accumulated cations in gelatin ionogel which leads to the thermovoltage decrease. It is also worth mentioning that the stabilized *V_oc_* of the NCTECs also showed an obvious increase when EMIM:DCA loading increased from 20 to 80 wt.% (Figure 2g), which can be attributed to the ion accumulation‐induced Seebeck enhancement.^[^ ^16^, ^21^ ^]^ The *I_sc_* curves of NCTECs with gelatin ionogel with different ionic liquid (IL) contents were measured immediately after thermovoltage reached stabilized values at *ΔT* = 1 K. (Figure 2b–e). In contrast to the steady current generated by either neat CPNF or NCTEC with 20 wt.% of ILs, the *I_sc_* exhibited a significant increase for NCTEC with 80 wt.% of EMIM:DCA with peak short‐circuit current density (*J_sc_*) improved from 0.06 to 2.31 A m^−2^ (Figure 2h,i). More importantly, NCTEC with 80 wt.% IL content exhibits only a slow decay over 2 h from 2.44 to 0.79 µA under constant heat supply, unlike the fast current decay occurring in the normal ionic TE capacitor discharging process.^[^ ^9^, ^10^, ^22^ ^]^ In addition to the thermovoltage and current output performance, the conductivity of the NCTEC was found to decrease to 17.7 S m^−1^ compared to 47.6 S m^−1^ of neat CPNF.

### 2.3 TE Performance Optimization of NCTEC

**Figure 3:**
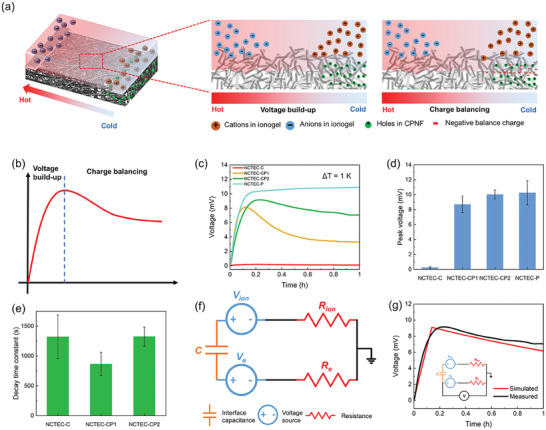
a,b) Schematic thermovoltage profile and mechanisms of an NCTEC under constant temperature gradient: i) voltage build‐up stage, and ii) charge balancing stage; c) The *V_oc_* profiles of the NCTECs made from gelatin ionogel (ILs loading 80 wt.%) and different electronic or dielectric layers; d) The peak *V_oc_* and e) fitted decay time constant of the NCTECs with different electronic or dielectric layers; f) Proposed equivalent circuit for NCTECs (*V_ion_*, *R_ion_*, *V_e_*, *R_e_* and *C* represent the ionic thermovoltage, ionic resistance of gelatin ionogel, electronic thermovoltage, electronic resistance of CPNF, and the interface capacitance in between, respectively); g) The measured and simulated thermovoltage profile using the proposed equivalent circuit in Figure 3f.

As schematically shown in **Figure** **3**a,b, the thermovoltage profile of an NCTEC can be divided into two stages. At the initial stage when *ΔT* starts to increase from 0, the hole accumulation in CPNF and cation/anion accumulation in gelatin ionogel in the lateral direction at the cold and hot end of NCTEC initiate due to their e‐TE and i‐TE behaviors, which leads to a rise in thermovoltage (voltage build‐up stage). As electronic charge carriers in CPNF and ionic charge carriers in gelatin ionogel cannot transport across the i/e interface, a capacitive interface similar to electrochemical double layers is established, leading to the charge compensation at i/e interface (Figure S7, Supporting Information). However, owing to the large interface capacitance, the voltage drop from charge balancing is much slower than the increase by the Soret effect and Seebeck effect.^[^ ^12^, ^15^ ^]^ Therefore, at this stage, the thermovoltage of NCTEC showed an increasing trend with ionic and electronic thermovoltage accumulation, while the large peak thermovoltage is also attributed to the very slow charge compensation rate with significantly enlarged interface capacitance between the i/e interface of NCTEC. When the temperature becomes stable, the thermovoltage gradually stops increasing, while the charge compensation continues, resulting in the decrease in thermovoltage (charge balancing stage). The existence of the balancing negative charges was further confirmed by the observed negative voltage of NCTEC from the delayed charge re‐balance when the temperature gradient was removed (Figure S8, Supporting Information).

To further optimize the TE performance of NCTEC, we subsequently investigated the thermovoltage profiles of NCTECs with different designs. NCTECs with pure single‐walled CNT (SWCNT), CPNF, and pure PLA nanofibrous fabric as e‐TE materials and 80 wt.% EMIM:DCA gelatin ionogel as i‐TE materials were fabricated which were denoted as NCTEC‐C, NCTEC‐CP1, NCTEC‐CP2, and NCTEC‐P (Table S2, Supporting Information). First, the peak thermovoltage of NCTEC is strongly correlated to the resistance of selected e‐TE materials (*R_e_*) when the resistance of ionogel (*R_i_*) was kept constant. As shown in Figure 3c, the NCTEC‐C with pure SWCNT film as e‐TE material exhibited the lowest thermovoltage profile and a peak *V_oc_* of 0.25 mV, which mainly attributed to the fast negative charge balance in SWCNT layer with an extremely low resistance of 0.9 Ω. Meanwhile, NCTEC‐CP using CPNF as e‐TE materials exhibited high peak *V_oc_* of 8.7 and 10.0 mV in both NCTEC‐CP1 with *R_e_* of 2.1 kΩ and NCTEC‐CP2 with *R_e_* of 3.35 kΩ (Figure 3d), owing to the slower migration of charge in high‐resistance CPNF to balance the interface capacitive effect. We have also tested the thermovoltage profile of NCTEC‐P with pure PLA nanofibrous fabrics to substitute e‐TE materials. With extremely high resistance (>40MΩ) of PLA fabrics, NCTEC‐P exhibited the highest peak *V_oc_* of 10.5 mV (Figure 3d), where charge carriers hardly transported to i/e interface to balance the thermovoltage generated from ionogel. Therefore, the NCTEC‐P is more like an ionic TE capacitor, where no depletion of voltage was observed over time, but the *I_sc_* was much lower than NCTEC‐CP (Figure S9, Supporting Information).

Another crucial parameter that affects the TE performance of NCTEC is the thermovoltage decay time constant (*τ = CR_e_*) in the charge balancing stage, which is sensitive to both interface capacitance and resistance of e‐TE materials. A larger *τ* indicates slower voltage decay, which can lead to higher TE performance. As shown in Figure 3e and Figure S10 (Supporting Information), by fitting the *V_oc_* to time curves using the exponential function, the *τ* of NCTEC‐CP1 and NCTEC‐CP2 are obtained of 866 and 1323 s, both of them are much higher than the *τ* of other i/e TECs reported previously (40–300 s) (Table S3, Supporting Information).^[^ ^15^, ^16^ ^]^ It can be mainly ascribed to the extremely high interface capacitance between CPNF and gelatin ionogel from large interface surface area and optimal resistance of CPNF in NCTEC‐CP.

To further study the significant effect of interface capacitance on the TE performance of NCTEC, we have designed a hybrid i/e TEC by covering gelatin ionogels with 80 wt.% EMIM:DCA loading onto hot‐pressed CNT/PLA composite films for minimized i/e contact area. Furthermore, to avoid the possible influence of *R_e_* on TE performance, the hot‐pressed CNT/PLA composite film with similar *R_e_* as the e‐layer was utilized. To experimentally confirm the difference in surface area of CPNF and hot‐pressed CNT/PLA composite films, atomic force microscopy (AFM) was conducted. AFM 2D and 3D images revealed that the hot‐pressed CNT/PLA composite film was much smoother than CPNF with smaller roughness (Figure S11 and Table S4, Supporting Information). As a consequence, a smaller i/e interface contact area and hence much lower interface capacitance are expected between gelatin ionogel and hot‐pressed CNT/PLA composite film. The TE performance of the hot‐pressed CNT/PLA‐gelatin ionogel hybrid TE converter was subsequently tested. As demonstrated in Figure S12 (Supporting Information), both peak *V_oc_* and *τ* values of the hot‐pressed CNT/PLA‐gelatin ionogel hybrid TE converter were much lower than the NCTEC‐CP2, indicating that the significant impact of interface capacitance on the TE performance enhancement of NCTEC.

Furthermore, an equivalent circuit is proposed for NCTECs, where *V_ion_*, *R_ion_*, *V_e_*, *R_e_*, and *C* represent the ionic thermovoltage, ionic resistance of gelatin ionogel, electronic thermovoltage, electronic resistance of CPNF, and the interface capacitance in between, respectively (Figure 3f). The measured thermovoltage profile of NCTEC‐CP2 over 1 h was then simulated with this equivalent circuit using the component values stated in Note S2 (Supporting Information). The simulated thermovoltage profile with the proposed equivalent circuit well matched the experimental results (Figure 3g). The effects of *R_e_* and *C* on peak *V_oc_* and *V_oc_* at 1 h (*V_1h_*) of the NCTEC were also investigated via simulation of the proposed equivalent circuit with different *R_e_* and *C* values. As exhibited in Figure S13 (Supporting Information), the peak *V_oc_* and *V_1h_* both increase with the increasing *R_e_* which is consistent with our experiment values. It is worth mentioning that the simulated peak *V_oc_* and *V_1h_* values increase with larger interface capacitance, therefore the voltage decay over 1 h (the voltage difference between peak *V_oc_* and *V_1h_*) is minimized. These results proved the significance of our structural design of NCTEC with large interfacial surface area to improve the interface capacitance and hence overall TE performance.

Subsequently, we have investigated the influence of heating rate on the thermovoltage profile of NCTEC‐CP2. As shown in Figure S14a (Supporting Information), when the heating rate slowed down from 0.7 to 0.07 K min^−1^, the increasing rate of *V_oc_* of NCTEC also slowed down. However, the peak *V*_oc_ values are not much affected by heating rates, which is different from the results of previously reported i/e TECs with decreased peak *V*_oc_ when the heating rate decreased.^[^ ^15^, ^16^ ^]^ For these i/e TECs, the decreased peak *V*_oc_ was mainly ascribed to the slower ion accumulation rate as the heating rate became slower, while the voltage drop by charge balancing between i/e interface was fast due to the small *R_e_* and interface capacitance. However, for NCTEC, the voltage decay contributed by charge balancing between i/e interface was largely slowed down owing to the large interface capacitance and *R_e_*. Therefore, the peak *V*_oc_ is not much affected by different heating rates. Apart from the experimental data, the simulated peak *V*_oc_ values as a function of heating rate were demonstrated in Figure S14b (Supporting Information), which is consistent with the experimental data.

### 2.4 Power Output Performance and Stability of NCTEC

**Figure 4:**
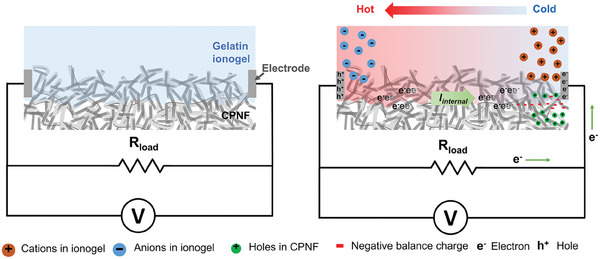
Schematic diagram of NCTEC connected to external load at the initial state and working principle after applying *ΔT*.

**Figure 5:**
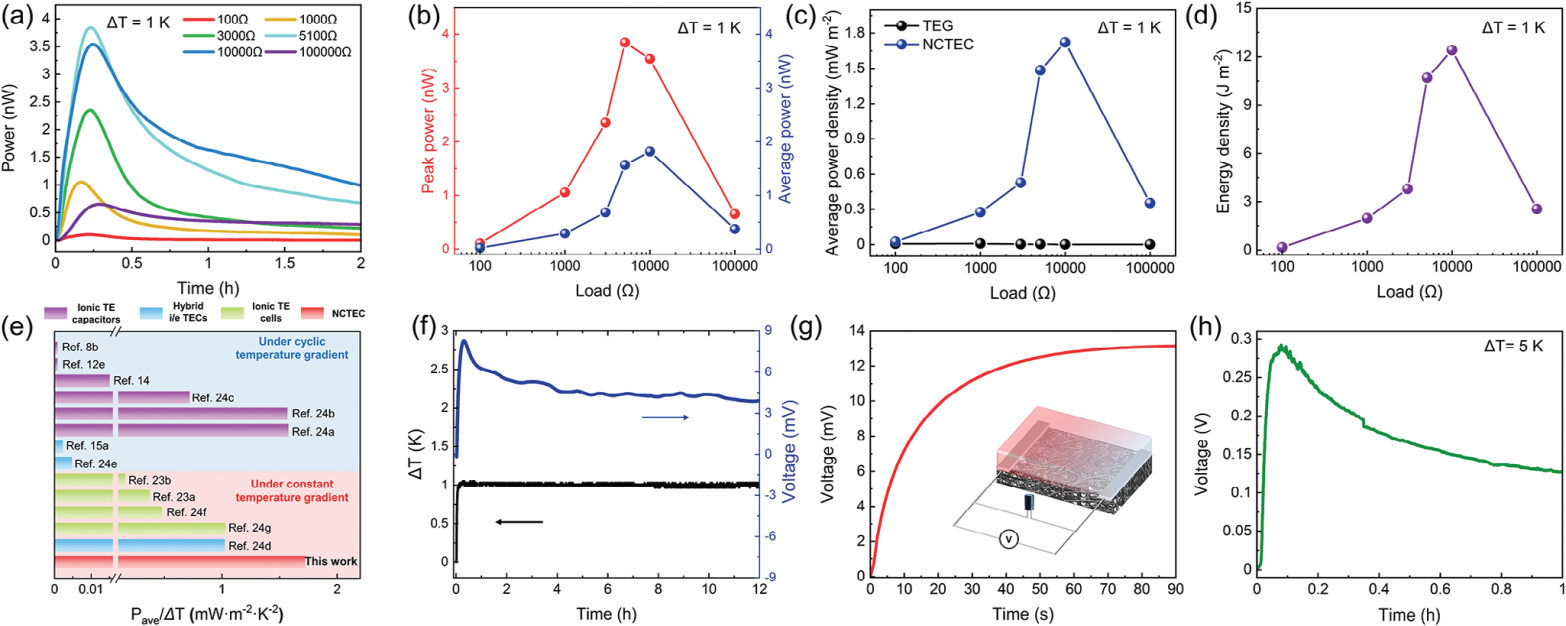
a) Output power to time curves of the NCTEC when connected to different external loads after applied with continuous temperature gradient of 1 K; b) The peak output power and 2‐h average output power as a function of different external load resistance connected; c) Comparison of the average output power density by the NCTEC and TEG made from CPNF under constant ΔT = 1 K for with various of external load resistance in 2 h; d) the 2‐h energy density (*E_2h_*) with different external loads connected when applied with continuous temperature gradient of 1 K; e) Comparison of average output power density to *ΔT^2^* of NCTEC with the reported values of other TE devices in literatures; f) *V_oc_* profile of the NCTEC under a continuous *ΔT* = 1 K over 12 h; g) Charging curve of a NCTEC for a 0.47 mF supercapacitor after being applied with 1 K temperature difference for 1 h; h) The *V_oc_* profile of a TE energy harvesting device made from 16 NCTEC modules connected in series under temperature difference of ≈5 K.

**Figure** **4** depicts the proposed working principle of NCTEC connected to external load. After applying *ΔT* to NCTEC, the thermovoltage gradually increases due to the ion accumulation of gelatin ionogel and hole migration of CPNF, while simultaneous negative charge balancing occurs at the i/e interface. Meanwhile, with the external load connected, electrons and holes can flow toward two electrodes to balance the potential generated and form an external electric current. Owing to our unique design of i/e hybrid structure, the output voltage of the NCTEC can realize slower decay compared with the ionic TE capacitors charging stage as the counter electrons from the external circuit to neutralize the potential between the electrodes can pass through the CPNF as internal electric current (*I_internal_*) and transport into the other electrode. Therefore, the NCTEC can generate higher output current and power continuously under a constant temperature gradient, which is consistent with the output current characteristic curves in Figure 2, where the output current and current density of the NCTEC were greatly enhanced compared to that of CPNF. The actual power output performance of the NCTEC under constant temperature difference was further characterized using NCTEC‐CP2 because of its higher thermovoltage and slower voltage decay. The temperature and output voltage profiles of NCTEC were recorded when heated up to a temperature gradient of 1 K with the external load connected throughout the testing period of 2 h (**Figure** **5**a; Figure S15, Supporting Information). The output voltage exhibited peak values when the heater was turned on for ≈15 min in all scenarios. Afterward, output voltage showed slow decay to relative stable values that are all above 0, which means that our NCTEC can maintain stable output under constant heating conditions. In contrast, the output behaviour of ionic TE capacitors is discontinuous where its output voltage rapidly decay to 0 in several minutes of time. The output power (*P*) is calculated by *P* = *U_output_* ^2^/*R_external_*, where *U_output_* is the output voltage and *R_external_* is the external load resistance. When the NCTEC was connected to an external loading of 5100 Ω, the peak output power reached a maximum of 3.85 nW (Figure 5b). Another key device performance evaluation parameter is the continuous output power density (e.g., 1 h or 2 h).^[^ ^23^ ^]^ For NCTEC, the maximum 2‐h average output power of 1.82 nW was obtained with 10 kΩ external load (Figure 5b), which is almost 560 times of that generated by the TEG made from CPNF (Figure 5c). The highest 2‐h energy density (*E_2h_*) approached 12.4 J m^−2^ with only 1 K of temperature gradient (Figure 5d). In addition, the output voltage and power profile of NCTEC‐CP2 with different load resistance after being thermal charged at a temperature gradient of 1 K for 3 h were also characterized (Figure S16, Supporting Information). Different from the previous power output scenario, the output voltage of the NCTEC after thermal charging exhibited the highest value right after the external load connection and showed similar slow voltage decay over a period of 2 h. In this case, the maximum instantaneous output power of 15.0 nW was obtained when connected to an external load of 1000 Ω. The maximum 2‐h average output power was 1.34 nW with a load resistance of 10 kΩ. For the convenience of comparison to the literature‐reported output performance, we use the output power density normalized by squared temperature gradient (*P_ave_/ΔT^2^*) to eliminate the contribution of temperature on output power density. As shown in Figure 5e and Table S5 (Supporting Information), the maximum *P_ave_/ΔT^2^* of our NCTEC is 1.72 mW m^−2^ K^−2^, which is up‐to‐date the highest among the i‐TE and i/e hybrid TE converters ever reported.^[^ ^8^, ^12^, ^14^, ^15^, ^23^, ^24^ ^]^

The long‐term stability of NCTEC was further examined by recording the thermovoltage profile under 1 K of temperature difference over 12 h. As shown in Figure 5g and Figure S17 (Supporting Information), both *V_oc_* and *J_sc_* were kept stable for more than 12 h. It could also be easily reactivated when the temperature gradient was removed and worked steadily over consecutive days. (Figure S18, Supporting Information). In order to demonstrate the capability of our NCTEC to power an external device, we connected a thermally charged NCTEC at ΔT of 1 K to a 0.47 mF capacitor. The voltage of the capacitor rapidly increased to the thermovoltage generated by the NCTEC in no more than 90 s, indicating the capability of NCTEC to charge a capacitor (Figure 5h). A 16‐leg of NCTEC connected head‐to‐tail in series with silver electrodes was further assembled (Figure S19, Supporting Information). The distance between the silver electrodes inside the NCTEC was increased to 8 mm for the convenience of electrode connection. Under a temperature difference of 5 K, the peak *V_oc_* of the device could rise to over 0.3 V and decay to 0.15 V after 1 h. The high voltage generated provides an opportunity for NCTEC to be used in practical applications such as wearable electricity generation from body heat harvesting.

## 3 Conclusion

In this study, high power output i/e hybrid NCTEC with continuous power generation was developed by the rational i/e interface engineering and the electrical resistance optimization of e‐TE materials. Based on the electrospray‐on‐electrospinning process, CPNF was readily prepared for integration with gelatin ionogel to form a hybrid i/e heterostructure. The high surface area of CPNF amplifies the interfacial capacitive effect between i/e heterostructure of the TE converter. Furthermore, the judicial manipulation of resistance of e‐TE materials plays a pivotal role in increasing the peak thermovoltage via minimizing charge balancing, resulting in a substantial increase in both output voltage and current over a long‐term steady temperature gradient. As a result, a record‐high output power density normalized by the squared temperature gradient (*P_ave_/ΔT^2^*) of 1.72 mW m^−2^ K^−2^ and a 2‐h energy density (*E_2h_*) of 12.4 J m^−2^ with only 1 K of temperature gradient are achieved. Our NCTEC also shows good output stability to operate continuously over 12 h and reusability for several consecutive days under operate‐rest‐operate working mode. Our work provides a novel and effective strategy to boost the power output and realize the continuous operation of hybrid i/e TE converters under a constant temperature gradient toward practical use.

## Conflict of Interest

The authors declare no conflict of interest.

## Supporting information



Supporting Information

## Data Availability

The data that support the findings of this study are available from the corresponding author upon reasonable request.

## References

[1] L. Yang , Z.‐G. Chen , M. S. Dargusch , J. Zou , Adv. Energy Mater. 2018, 8, 1701797.

[2] a) R. Liu , Z. L. Wang , K. Fukuda , T. Someya , Nat. Rev. Mater. 2022, 7, 870;

[3] a) X.‐L. Shi , J. Zou , Z.‐G. Chen , Chem. Rev. 2020, 120, 7399; 32614171 10.1021/acs.chemrev.0c00026

[4] G. J. Snyder , E. S. Toberer , Nat. Mater. 2008, 7, 105. 18219332 10.1038/nmat2090

[5] a) S. Masoumi , S. O'Shaughnessy , A. Pakdel , Nano Energy 2022, 92, 106774;

[6] a) W. Li , S. Garcia , S. Wang , in Low‐Grade Thermal Energy Harvesting, (Ed.: S. Wang ), Woodhead Publishing, Sawston, Cambridge 2022;

[7] a) J. Chen , L. Zhang , Y. Tu , Q. Zhang , F. Peng , W. Zeng , M. Zhang , X. Tao , Nano Energy 2021, 88, 106272;

[8] a) S. L. Kim , J.‐H. Hsu , C. Yu , Org. Electron. 2018, 54, 231;

[9] a) E. D. Eastman , J. Am. Chem. Soc. 1928, 50, 283;

[10] a) Y.‐H. Pai , J. Tang , Y. Zhao , Z. Liang , Adv. Energy Mater. 2023, 13, 2202507;

[11] H. Wang , U. Ail , R. Gabrielsson , M. Berggren , X. Crispin , Adv. Energy Mater. 2015, 5, 1500044.

[12] a) Z. Liu , H. Cheng , H. He , J. Li , J. Ouyang , Adv. Funct. Mater. 2022, 32, 2109772; 10.1002/adfm.202200260 PMC9514151 36176721

[13] a) H. Cheng , Q. Le , Z. Liu , Q. Qian , Y. Zhao , J. Ouyang , J. Mater. Chem. C. 2022, 10, 433;

[14] C. Cho , B. Kim , S. Park , E. Kim , Energy Environ. Sci. 2022, 15, 2049.

[15] a) H. Cheng , J. Ouyang , Adv. Energy Mater. 2020, 10, 2001633;

[16] H. Cheng , S. Yue , Q. Le , Q. Qian , J. Ouyang , J. Mater. Chem. A. 2021, 9, 13588.

[17] S. Liu , M. Zhang , J. Kong , H. Li , C. He , Compos. Sci. Technol. 2023, 243, 110245.

[18] C. T. L. Kenry , Prog. Polym. Sci. 2017, 70, 1.

[19] a) A. Peigney , C. Laurent , E. Flahaut , R. R. Bacsa , A. Rousset , Carbon 2001, 39, 507;

[20] U. Ail , M. J. Jafari , H. Wang , T. Ederth , M. Berggren , X. Crispin , Adv. Funct. Mater. 2016, 26, 6288.

[21] Z. Fan , D. Du , X. Guan , J. Ouyang , Nano Energy 2018, 51, 481.

[22] D. Zhao , H. Wang , Z. U. Khan , J. C. Chen , R. Gabrielsson , M. P. Jonsson , M. Berggren , X. Crispin , Energy Environ. Sci. 2016, 9, 1450.

[23] a) Y. Li , Q. Li , X. Zhang , B. Deng , C. Han , W. Liu , Adv. Energy Mater. 2022, 12, 2103666;

[24] a) Q. Qian , H. Cheng , Q. Le , J. Ouyang , Adv. Funct. Mater. 33, 2303311;

