# Supplementary material for: Giant Power Output from Ionic/electronic Hybrid Nanocomposite Thermoelectric Converter Under Constant Temperature Gradient
Source: Adv Sci (Weinh). 2024 Nov 23;12(3):2406589. doi: 10.1002/advs.202406589 (PMC11744677; doi:10.1002/advs.202406589)
Supplement: Supplementary file 1 — Supporting Information [file ADVS-12-2406589-s001.docx]

**Supporting information**

**Giant Power Output from Ionic/electronic Hybrid Nanocomposite Thermoelectric Converter under Constant Temperature Gradient**

*Siqi Liu,^a1^ Mingxia Zhang,^a1^ Junhua Kong,^b^ Hui Li,^c^ Chaobin He^ab*^*

a. Department of Materials Science & Engineering, National University of Singapore, 9 Engineering Drive 1, 117574, Singapore.

b. Institute of Materials Research and Engineering, A*STAR (Agency for Science, Technology and Research), 138634, Singapore.

c. Hubei Key Laboratory of Plasma Chemistry and Advanced Materials, Hubei Engineering Technology Research Center of Optoelectronic and New Energy Materials, School of Materials Science and Engineering, Wuhan Institute of Technology, Wuhan, 430205, China.

**Supplementary Note S1:**

**Materials.** Polylactic acid (PLA, grade Ingeo 3051D, weight average molar mass (*Mw*): 160,000, PDI: 1.7, density: 1.25 g/cm^3^, with 96.5% L-lactide and 3.5% D-lactide) was purchased from Nature Works LLC. The SWCNT paste (TNNPS) was purchased from Time Nano. Hexafluoro isopropanol (HFP, >99 %), 1-ethyl-3-methylimidazolium dicyanamide (EMIM:DCA, ≥98 %) and gelatin (gel strength 300, type A) were purchased from Sigma-Aldrich. Ethanol (absolute, >99.8 %) was purchased from Fisher Scientific. Silver paint (Pelco® 16062) was purchased from Ted Pella.

**Fabrication of CNT/PLA nanofibrous composite fabrics (CPNF).** The CNT/PLA nanofibrous composite fabrics (CPNF) were fabricated via electrospray-on-electrospinning technique as reported in our previous work. ^[1]^ The PLA/HFP electrospinning solution (16 wt%) and CNT/DMF/ethanol electrospray solution were loaded into 22-gauge/17-gauge coaxial needle. The electrospray-on-electrospinning CPNF was obtained under a voltage of 25 kV applied between the needle and collector with designed solution feed rates by electrospinning machine (NANON-01A, MECC company). The total PLA/HFP solution volume for electrospinning was controlled at 3 ml. Afterwards, the CPNF were collected from the collector and vacuum dried at room temperature overnight. Neat PLA fabrics with respective electrospinning conditions were also prepared with a 22-gauge needle.

**Preparation of the gelatin ionogel.** First, the gelatin was dissolved in deionized (DI) water with a fixed weight ratio of gelatin to DI water at 1:9, and then the solution was heated and stirred at a speed of 300 rpm on a hot plate at 50 ⁰C for 30 min. After the gelatin was completely dissolved in the DI water, EMIM:DCA were added to the solution to obtain the designed mass ratio of EMIM:DCA to gelatin from 1:4, 2:3, 3:2 and 4:1. The resulting solution continued to be stirred under the same condition on a hot plate at 50 ⁰C for 30 min. A gelatin ionogel film was obtained by spin coating the final solution on the pre-cleaned glass substrate at 1800 rpm for 60 s and drying at room temperature for 24 hours.

**Preparation of NCTECs.** Two parallel silver electrodes were applied on the rectangular CPNF with 2mm width and 2 mm distance in between. Afterwards, the gelatin ionogel solution was uniformly drop casted on top and dried at room temperature for 24 hrs.

**Characterizations.** The scanning electron microscopy (SEM) observation was conducted on a Zeiss Sigma 300 SEM. The electrical conductivity *(σ*) of the CPNF and NCTEC was obtained following 4-point probe methods with Van de Pauw geometry using Keithley 2000 multimeter. The gelatin ionogel samples were sandwiched between two stainless steel electrodes to measure the impedance of gelatin ionogel with an IVIUMnSTAT electrochemical station. The ionic resistance (*R_i_*) was than obtained in terms of the intercept of the straight line on the abscissa in the Nyquist plot, and the ionic conductivity (*σ_i_*) was calculated from the following equation,

$$\sigma_{i}=\frac{l}{R_{i}A}$$

where l and A are thickness and area of the gelatin ionogel sample, respectively. The electronic Seebeck coefficient (*S_e_*) of the CPNF and ionic Seebeck coefficient (*S_i_*) of the gelatin ionogels were measured with a home-made Seebeck coefficient testing system as previously reported. The open circuit thermovoltage (*V_oc_*), short circuit current (*I_sc_*) and output voltage profiles were recorded with a Keithley DMM6500 digital multimeter at room temperature of 22 ⁰C and relative humidity (RH) of 78–81 % unless otherwise noted. The surface morphology and roughness results were obtained using Atomic Force Microscope (AFM) (Bruker Dimension ICON) in the standard tapping mode (in the air), with a scanning rate at 1 Hz and spot size of 35 × 35 µm. The TE behaviors simulation of NCTECs were conducted using a free analog electronic circuit simulator software (LTspice).

**Statistical Analysis** Standard analysis was used to calculate the average values and standard deviations. The average peak *V_oc_* and decay time constant values are presented in the form of “mean ± standard deviation”. The number of samples used for statistical analysis was typically three, unless otherwise specified. The analysis was performed in Origin Pro 2021.


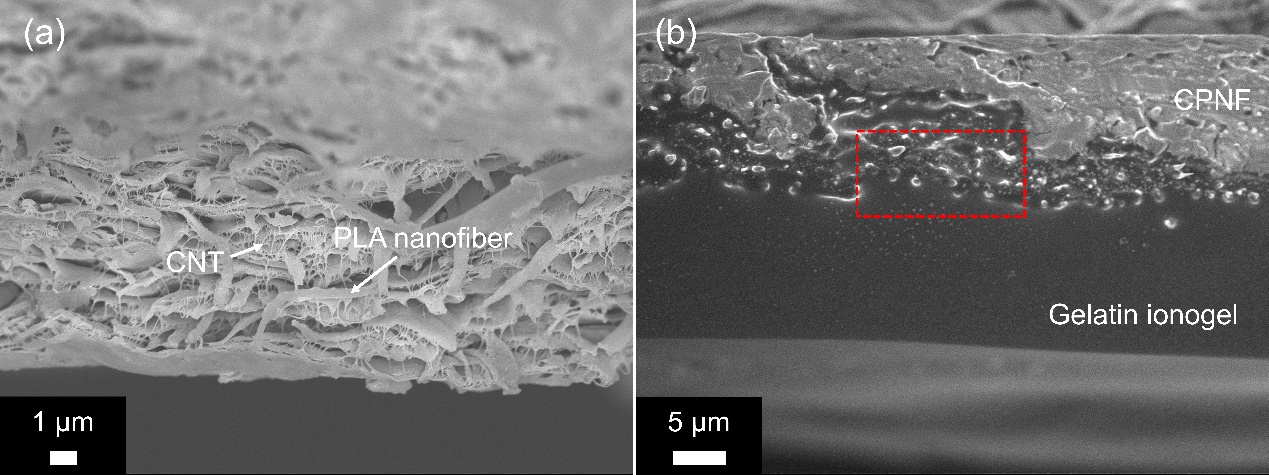


**Figure S1.** Cross-sectional SEM images of (a) CPNF and (b) NCTEC (the i/e interpenetrating region indicated in red dashed rectangle box).


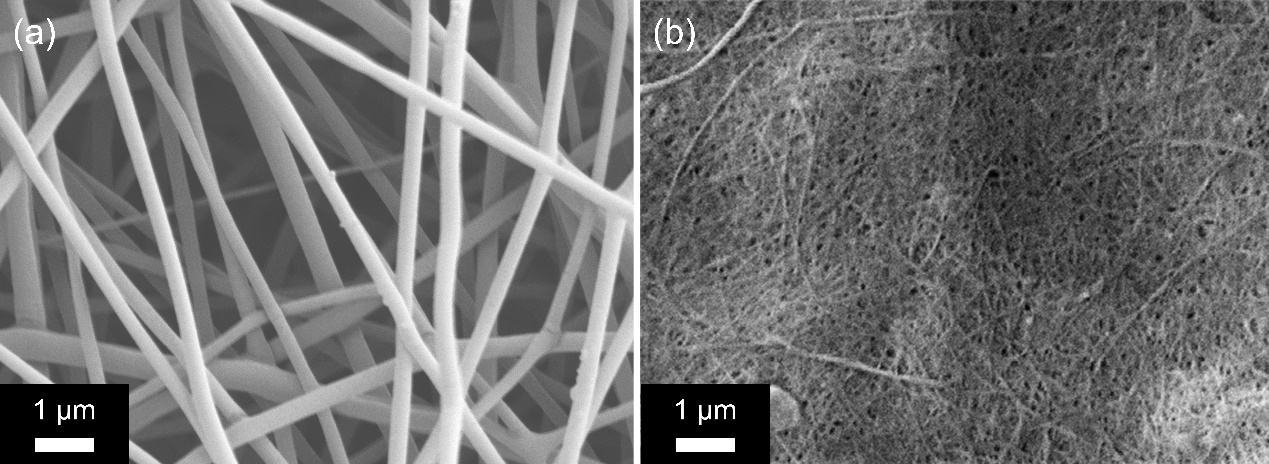


**Figure S2**. SEM images of (a) neat electrospun PLA nanofibers and (b) neat CNT film.


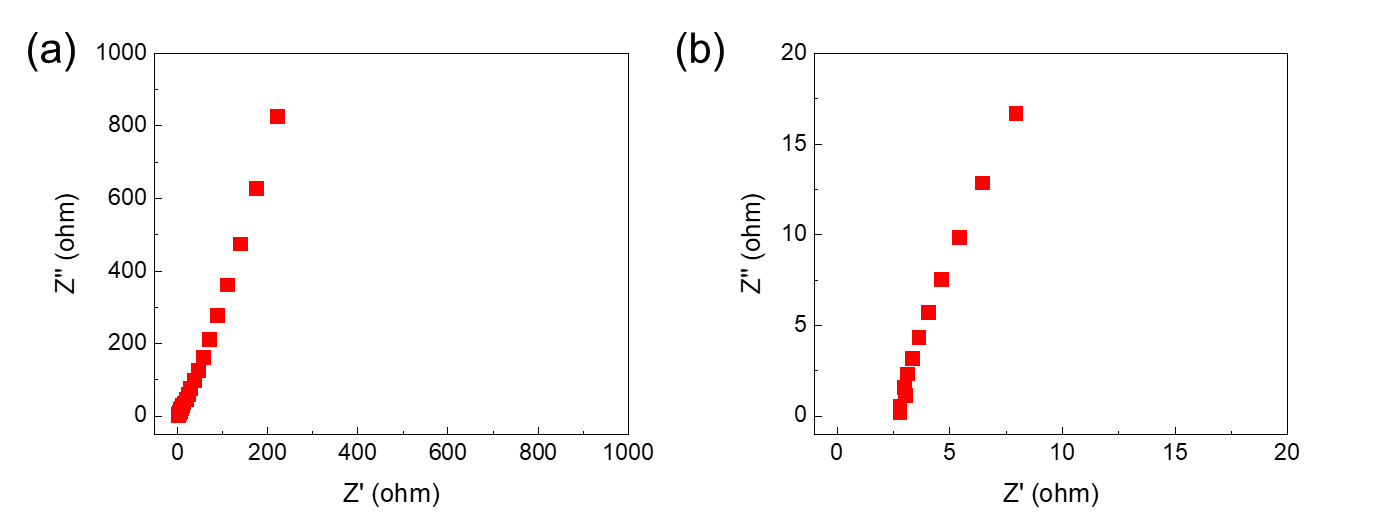


**Figure S3** The Nyquist plot of gelatin ionogel with 80 wt% of EMIM:DCA loading (a) whole testing frequency from 0.1 Hz to 100 kHz; (b) the plot near high frequency range.


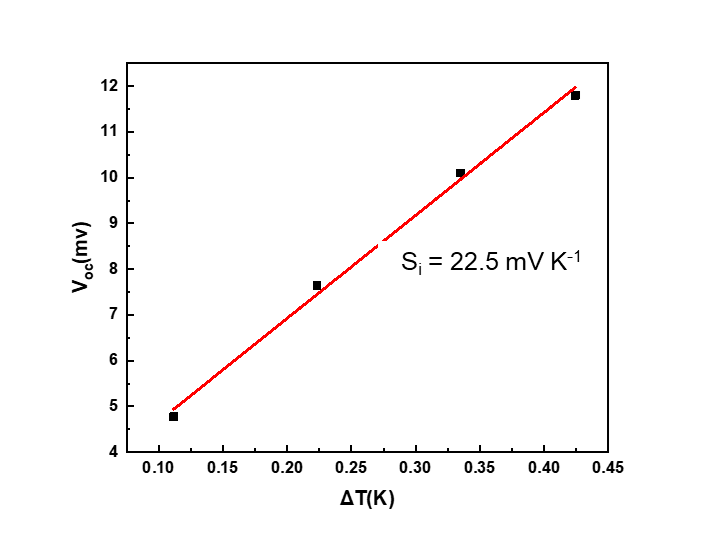


**Figure S4** The ionic Seebeck coefficient of a gelatin ionogel with the EMIM:DCA loading of 80 wt.%.


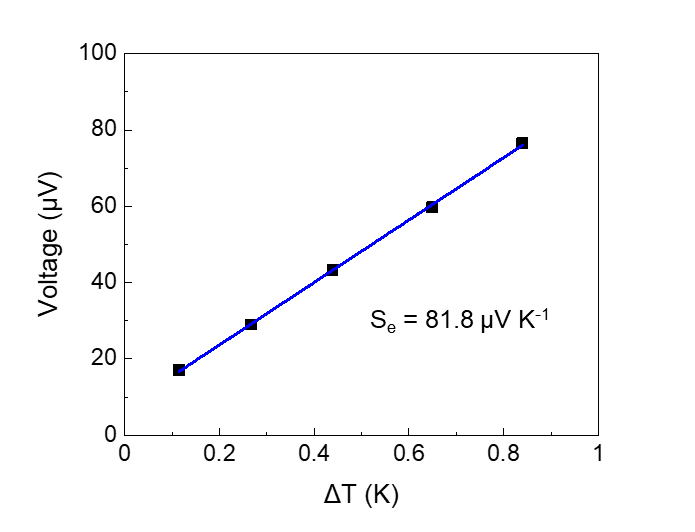


**Figure S5** The electronic Seebeck coefficient of an CPNF film.

**Figure S6** The temperature gradient profile applied when measuring the *V_oc_* and *I_sc_* of NCTEC with different IL loadings.


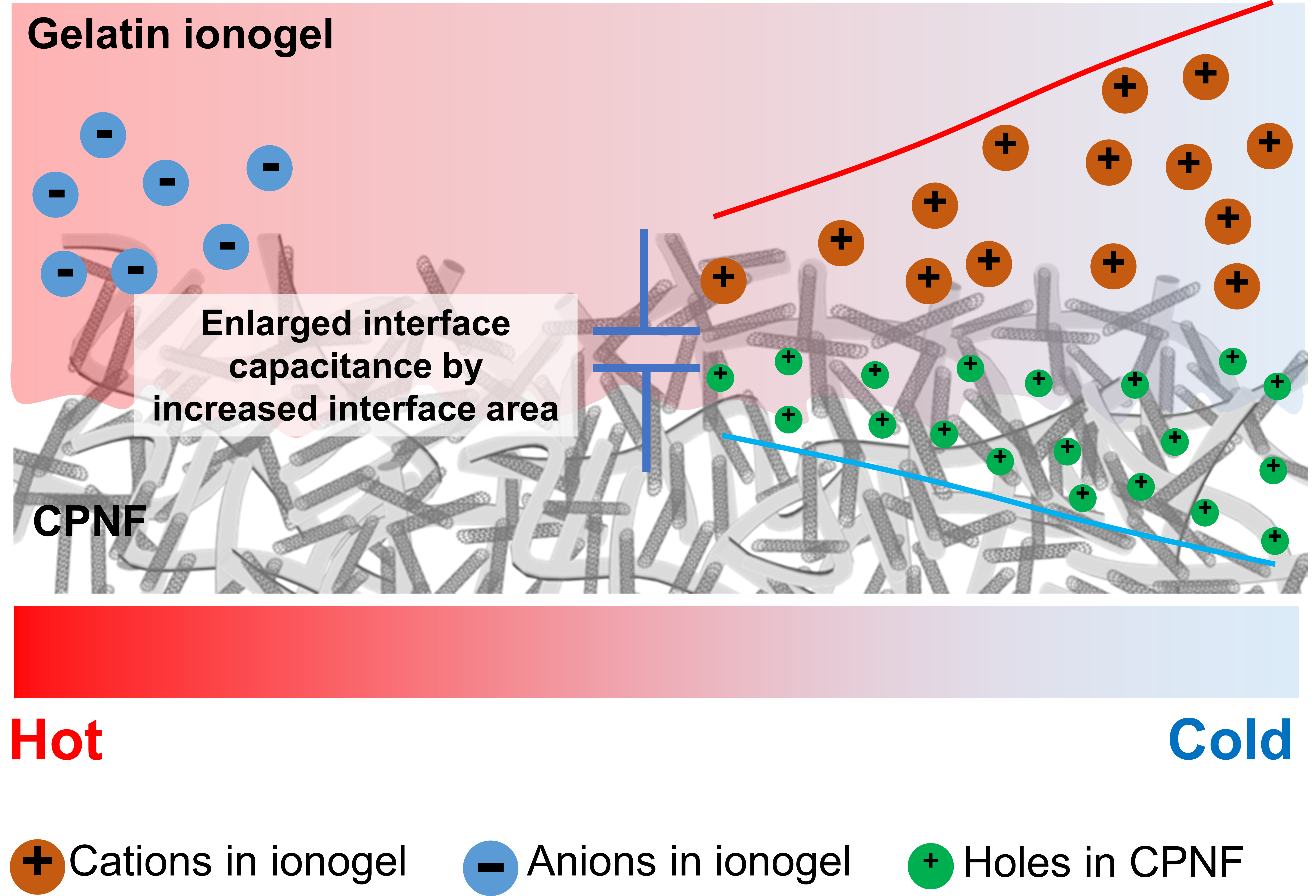


**Figure S7** Schematic diagram and mechanism sketch of NCTEC integrated with CPNF and gelatin ionogel under temperature gradient.


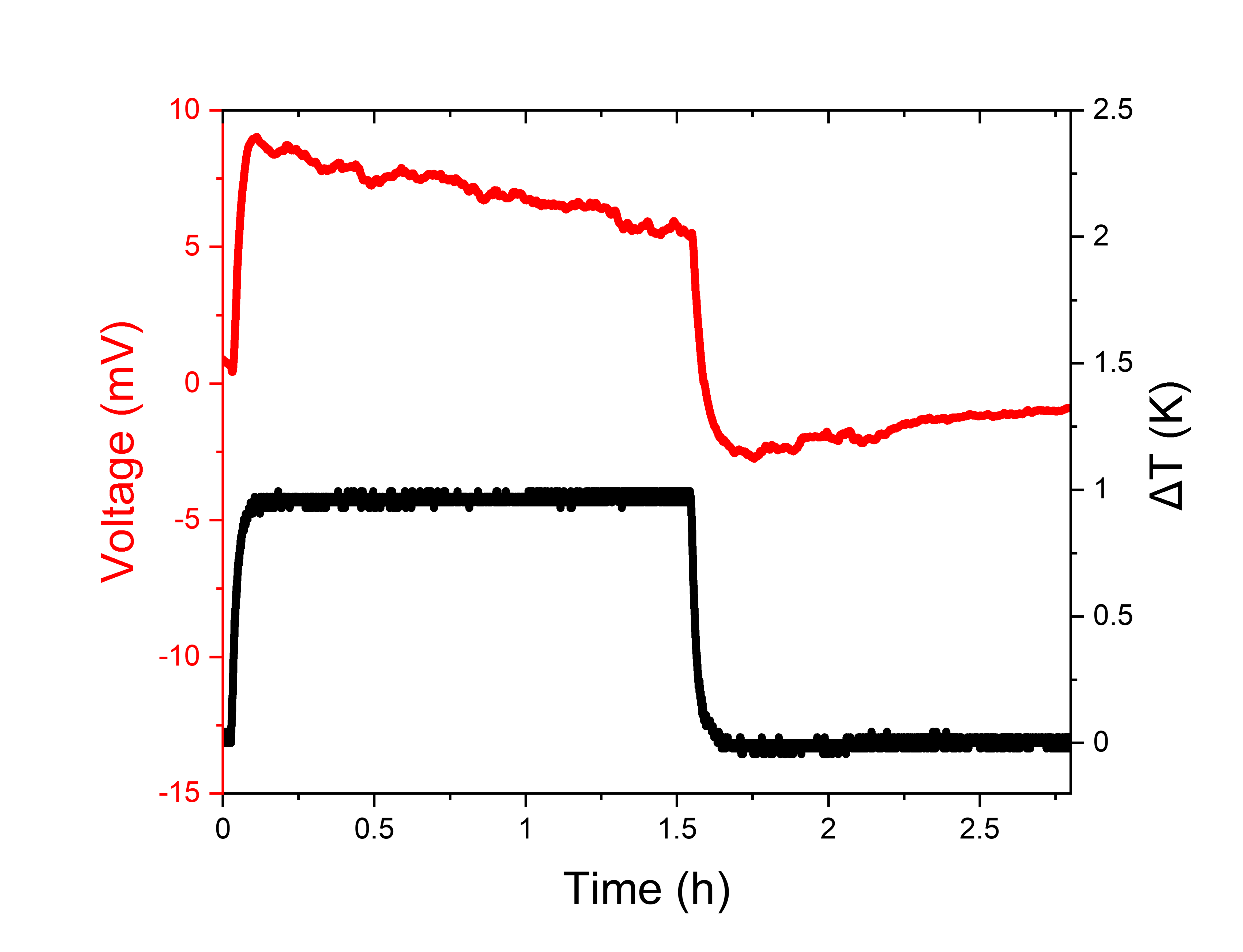


**Figure S8.** The thermovoltage profile of NCTEC with on-off heating profile.


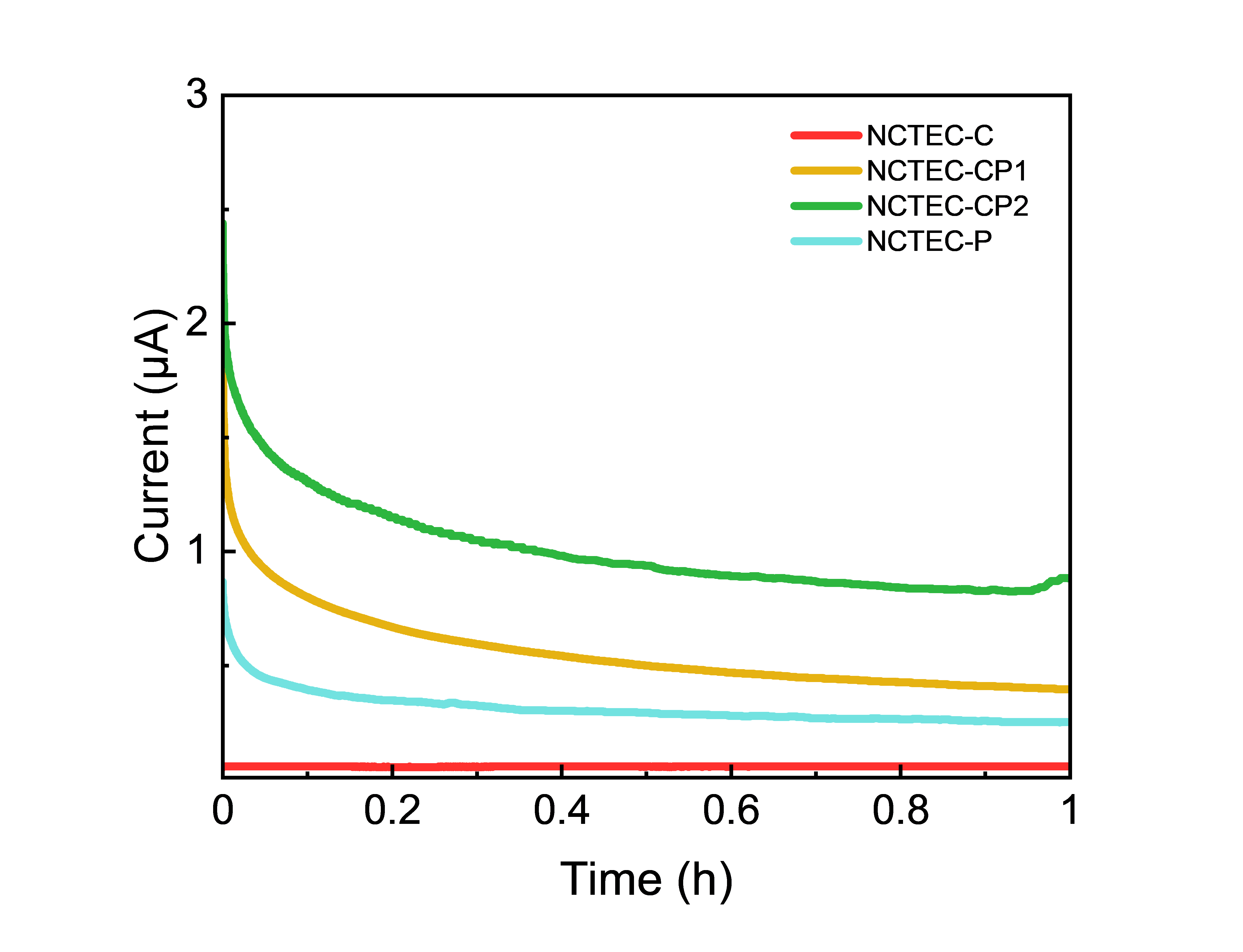


**Figure S9** The *I_sc_* curves versus time of NCTEC-C, NCTEC-CP1, NCTEC-CP2, and NCTEC-P after heating to a temperature difference of 1 K.


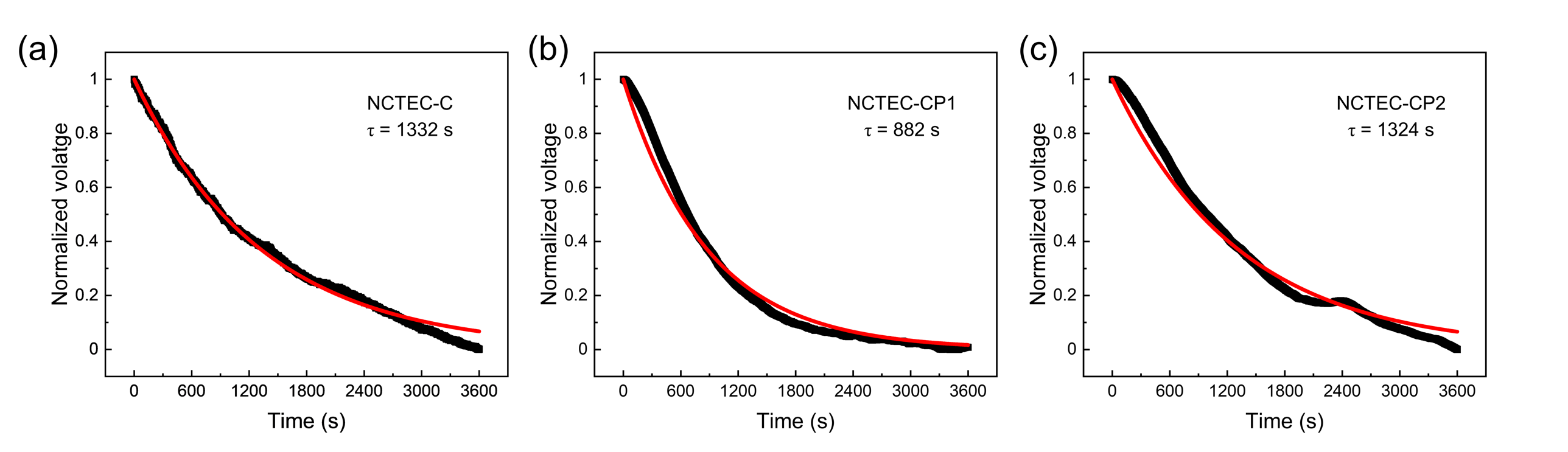


**Figure S10** The normalized thermovoltage profile and fitted curves (in red) of NCTEC-C, NCTEC-CP1 and NCTEC-CP2 using exponential function with decay time constant (τ).


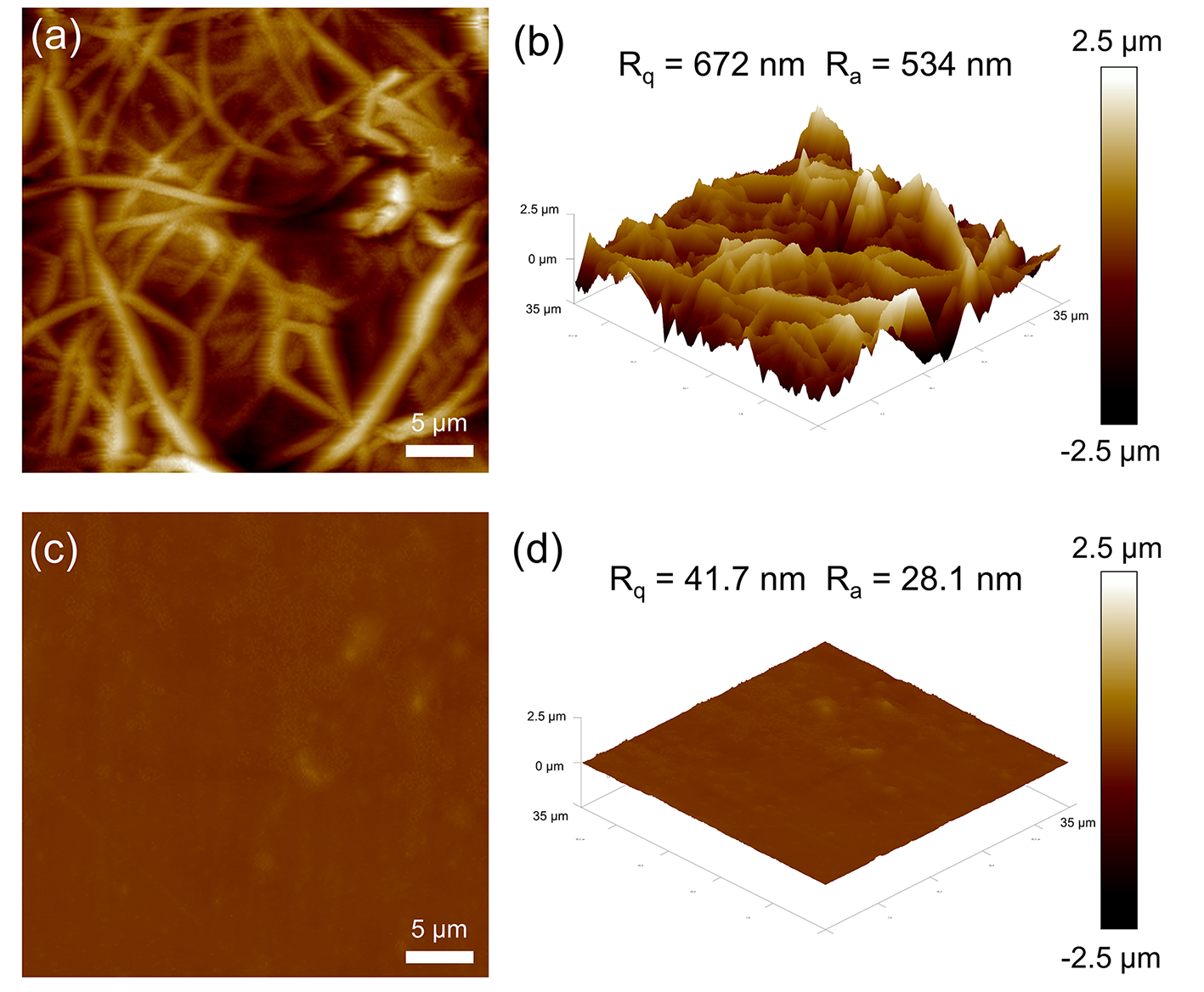


**Figure S11.** 2D and 3D AFM images of (a, b) CPNF and (c, d) hot-pressed CNT/PLA composite film.


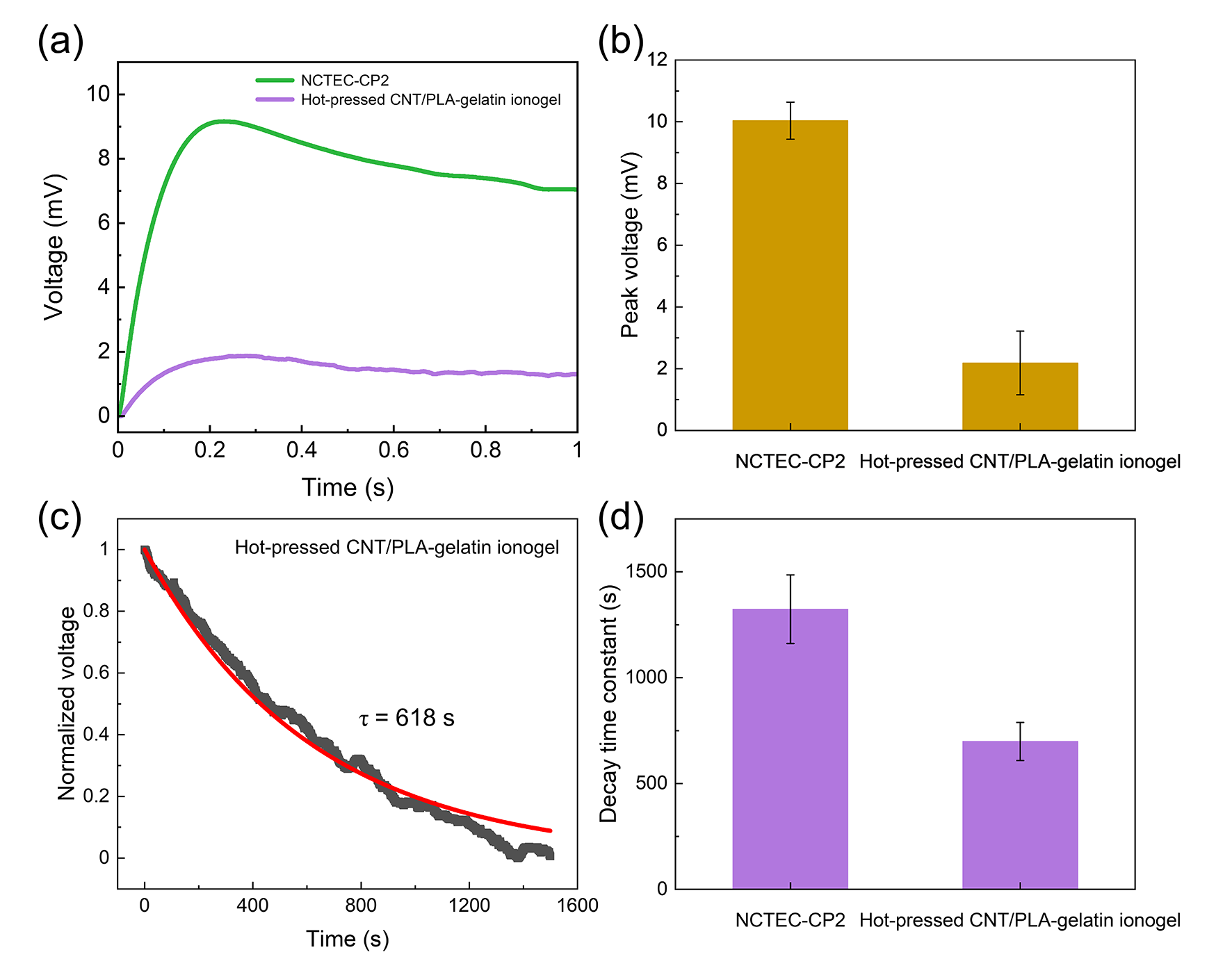


**Figure S12.** TE performance comparison between NCTEC-CP2 and hot-pressed CNT/PLA-gelatin ionogel hybrid i/e TECs

**Supplementary Note S2: Thermovoltage simulation of the NCTEC with proposed equivalent circuit**

The thermovoltage simulation of the NCTEC was determined with the equivalent circuit proposed in Figure 3f with following components values: electronic resistance (*R_e_*) of 3.35 kΩ and Seebeck coefficient of 81.8 μV K^-1^ of CPNF, ionic resistance (*R_i_*) of 4.5 kΩ and Seebeck coefficient of 21.9 mV K^-1^ of gelatin ionogel, the interfacial capacitance of 1 F. These values were chosen in terms of our measurement results.


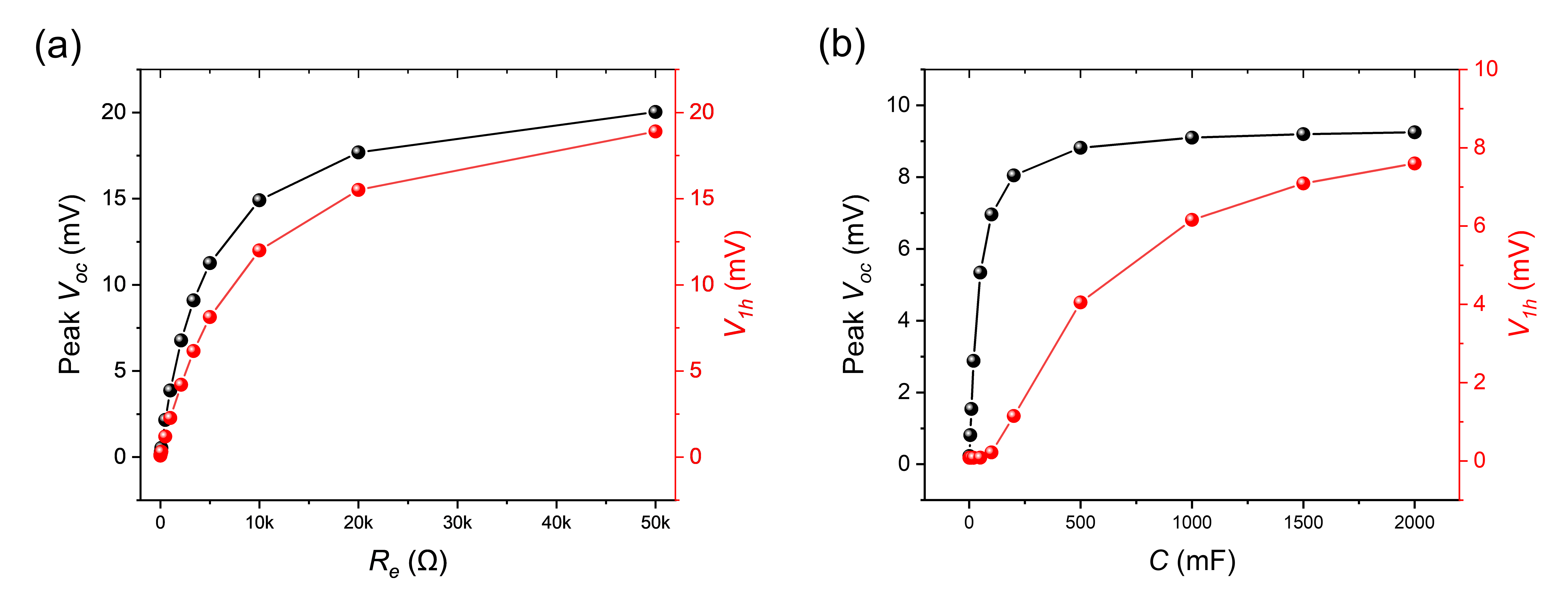


**Figure S13** Simulated peak *V_oc_* and *V_oc_* at 1 hour (*V_1h_*) with different (a) *R_e_* and (b) *C* with equivalent circuit proposed in Figure 3f.


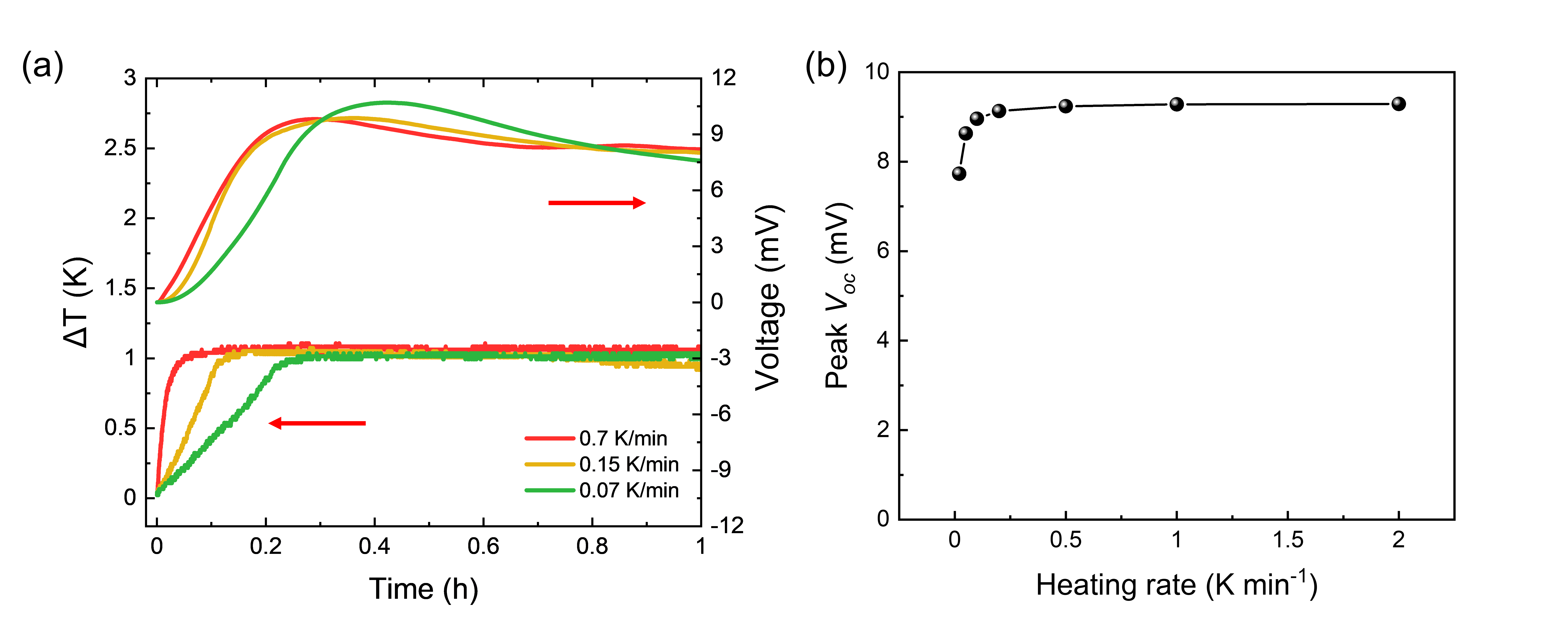


**Figure S14** The experimental thermovoltage profiles of NCTEC under different heating rates from 0.07 to 0.7 K min^-1^; (b) Simulated peak *V_oc_* with different heating rates.


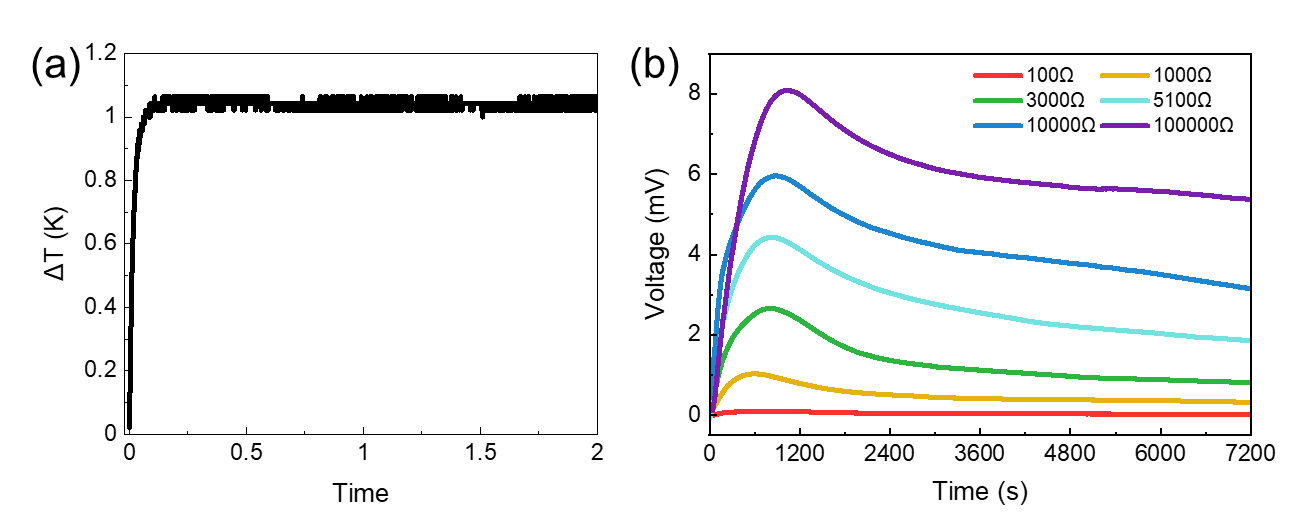


**Figure S15** (a) The heating profile and (b) output voltage of NCTEC with different load resistance when heated up to a temperature gradient of 1 K with external load connected throughout the testing period of 2 hours.


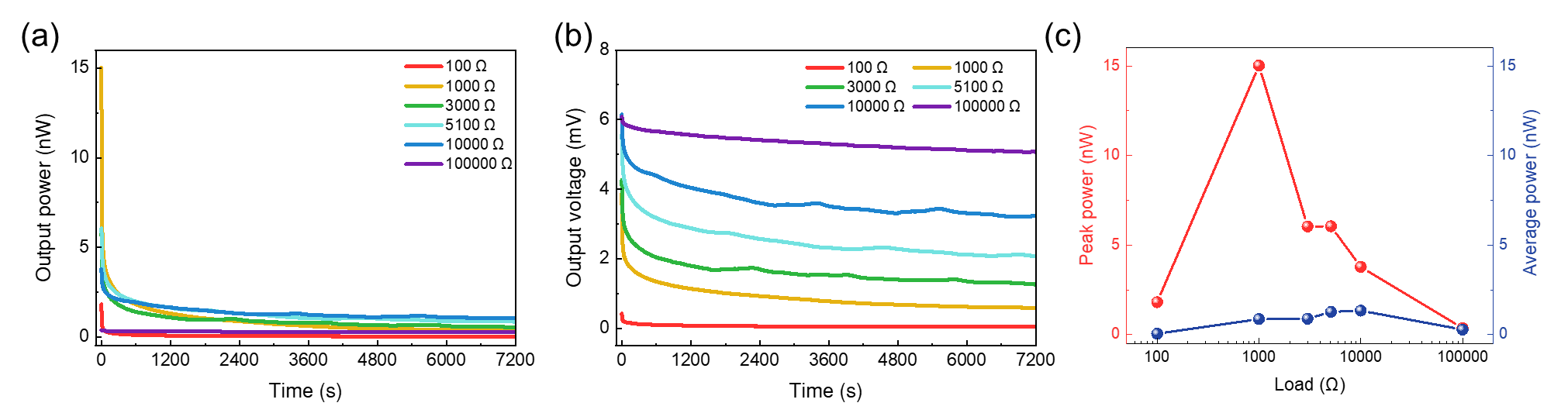


**Figure S16** The (a) output power and (b) output voltage profile of NCTEC with different load resistance after being thermal charged at a temperature difference of 1 K for 3 hours; (c) the maximum instantaneous output power the 2-hours average output power with NCTEC different external load resistance connected.

**Figure S17** The short-circuit current density profile of the NCTEC under a continuous ΔT = 1 K over 12 hours;


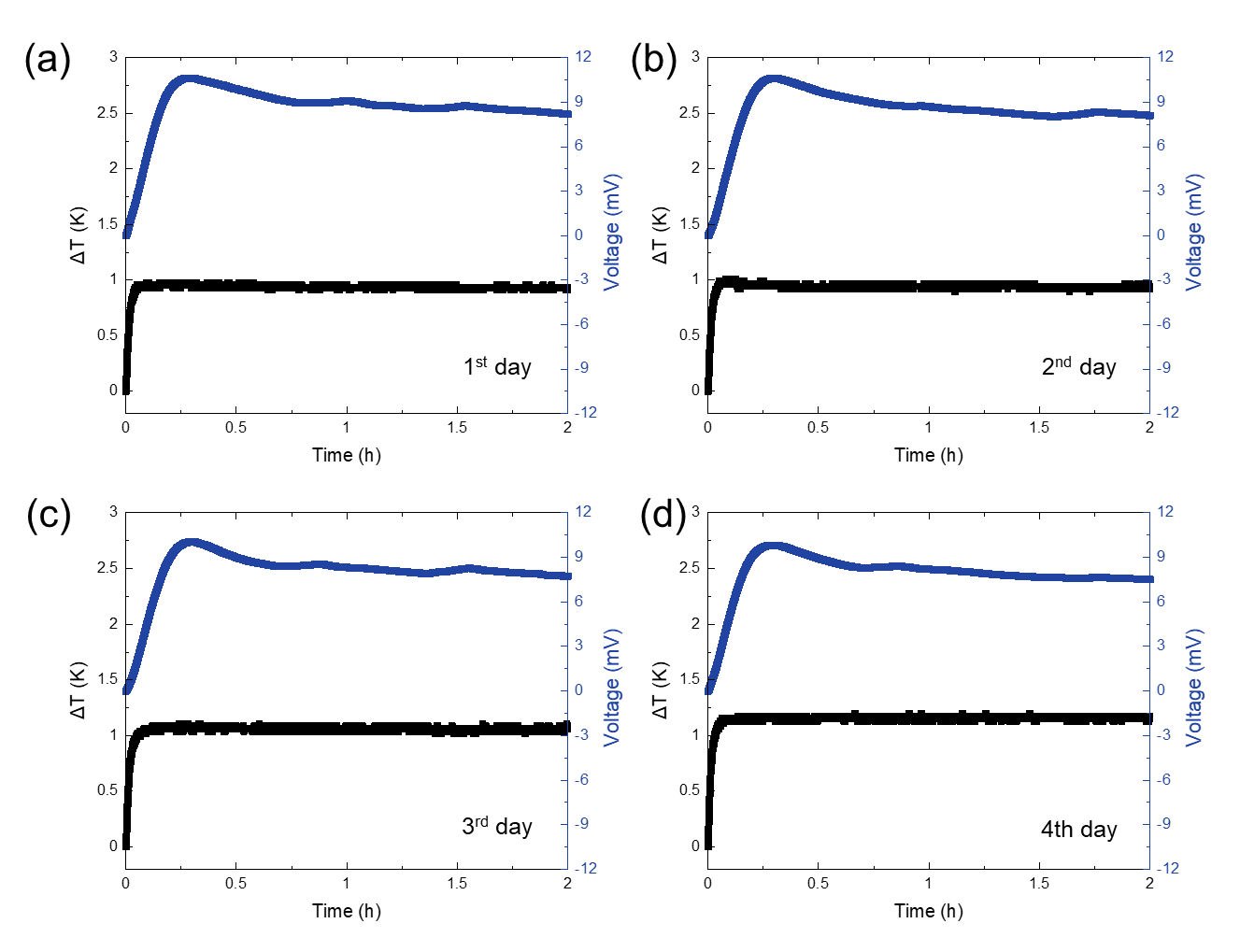


**Figure S18** The thermovoltage profile of NCTEC over several consecutive days. The (a) 1^st^ day, (b) 2^nd^ day, (c) 3rd day, and (d) 4^th^ day. The NCTEC was stored at room temperature with RH of 80%.

**
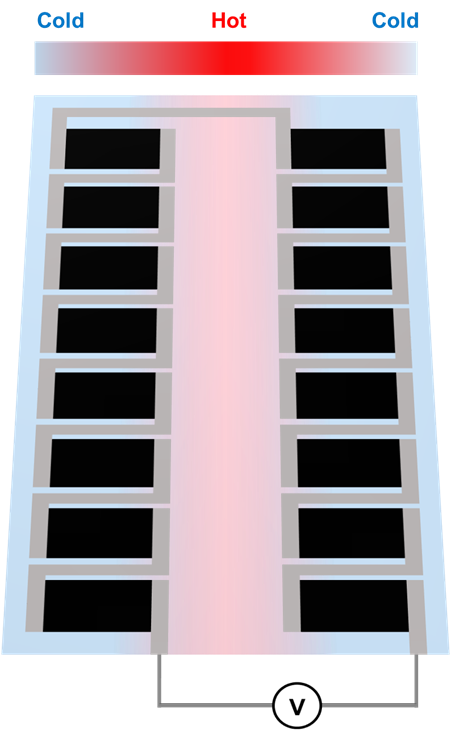
**

**Figure S19** The schematic illustration of a designed device which is consist of 16-leg of NCTEC connected head-to-tail in series with silver electrodes.

**Table S1.** The TE properties of CPNF and gelatin ionogel with 80wt % EMIM:DCA.

| Sample | *σ*  (S m^-1^) | *S*  (mV K^-1^) |
| --- | --- | --- |
| CPNF | 47.6 | 0.0818 |
| Gelatin ionogel with 80wt % EMIM:DCA | 0.60 | 21.9 |

**Table S2.** The design and properties of NCTEC with different e-TE materials.

| Sample | e-TE material | i-TE material | R_e_ (Ω) |
| --- | --- | --- | --- |
| NCTEC-C | SWCNT film | Gelatin ionogel with 80wt % EMIM:DCA | 0.9 |
| NCTEC-CP1 | CPNF | Gelatin ionogel with 80wt % EMIM:DCA | 2.1k |
| NCTEC-CP2 | CPNF | Gelatin ionogel with 80wt % EMIM:DCA | 3.35k |
| NCTEC-P | Electrospun PLA fabric | Gelatin ionogel with 80wt % EMIM:DCA | >40M |

**Table S3.** The decay time constant (τ) of NCTEC with different e-TE materials and i/e hybrid TE converters reported in literatures.

| Sample | NCTEC-C | NCTEC-CP1 | NCTEC-CP2 | PEDOT:PSS/  Ionogel HTEC^[2]^ | Bi_2_Te_3_/  Ionogel HTEC^[3]^ | SWCNT/gelatin ionogel MTEC^[4]^ |
| --- | --- | --- | --- | --- | --- | --- |
| Decay time constant (s) | 1321 | 866 | 1323 | 40-220 | 130-200 | 110-300 |

**Table S4.** The summary of *R_e_* and surface roughness of NCTEC and hot-pressed CNT/PLA-gelatin ionogel heterostructure.

| Sample | e-TE material | i-TE  material | *R_e_*  (Ω) | *R_a_*  (nm) | *R_q_*  (nm) |
| --- | --- | --- | --- | --- | --- |
| NCTEC-CP2 | CPNF | Gelatin ionogel with 80wt % EMIM:DCA | 3.35k | 534 | 672 |
| Hot-pressed CNT/PLA-gelatin ionogel | Hot-pressed CNT/PLA film | Gelatin ionogel with 80wt % EMIM:DCA | 2.95k | 28.1 | 41.7 |

**Table S5** Comparison of output power density normalized by squared temperature gradient (P_ave_/ΔT^2^) of NCTEC to the ionic TE devices and i/e hybrid TE converters reported in literature.

| TE device type | Materials | P_ave_  (mW m^-2^) | ΔT  (K) | P_ave_/ΔT^2^  (mW m^-2^ K^-2^) | Ref. |
| --- | --- | --- | --- | --- | --- |
| Ionic TE capacitors | PANI:PAAMPSA:PA | 2.38×10^-3^ | 1.7 | 8.2×10^-4^ | ^[5]^ |
|  | PVDF:HFP/EMIM:DCA | 8.4×10^-4^ | 0.6 | 2.3×10^-3^ | ^[6]^ |
|  | PEDOT:PAAMPSA:BS | 0.207 | 3.7 | 1.51×10^-2^ | ^[7]^ |
|  | SiO_2_/EMIM:DCA | 0.18 | 0.5 | 0.72 | ^[8]^ |
|  | Gelatin/EMIM:DCA | 14.12 | 3 | 1.569 | ^[9]^ |
|  | ZIF-8/EMIM:DCA | 14.2 | 3 | 1.578 | ^[10]^ |
| Ionic TE cells | Gelatin-KCl-FeCN_4_^-^ | ~24 | 8- | 0.375 | ^[11]^ |
|  | Gelatin-KCl-FeCN_4_^-^ | - | - | 0.16 | ^[12]^ |
|  | Gelatin/betane/[Fe(CN)_6_]^3-^ | 432.23 | 30 | 0.48 | ^[13]^ |
|  | Gelatin-m/n FeCN_4_^-^/3-x wt% Gr/ rGO/GO | - | 3 | 1.03 | ^[14]^ |
| Hybrid i/e TECs | PEDOT:PSS/PVDF：HFP/EMIM:DCA | 2×10^-4^ | 0.5 | 8×10^-4^ | ^[2]^ |
|  | PEDOT:PSS/PVDF：HFP/EMIM:DCA  /PEDOT:PSS/PEI-CNT/polyimde | 1.73×10^-3^ | 0.6 | 4.8×10^-3^ | ^[15]^ |
|  | CPP900-BMIM:Cl | 410 | 20 | 1.025 | ^[16]^ |
| **NCTEC** | **CPNF/gelatin/EMIM:DCA** | **1.72** | **1** | **1.72** | **This work** |

**References**

[1] S. Liu, M. Zhang, J. Kong, H. Li, C. He, *Compos. Sci. Technol.* **2023**, *243*, 110245.

[2] H. Cheng, J. Ouyang, *Adv. Energy Mater.* **2020**, *10*, 2001633.

[3] H. Cheng, Y. Liu, F. Cao, Q. Zhang, J. Ouyang, *Chem. Eng. J.* **2022**, *450*, 138433.

[4] H. Cheng, S. Yue, Q. Le, Q. Qian, J. Ouyang, *J. Mater. Chem. A* **2021**, *9*, 13588.

[5] Z. A. Akbar, J.-W. Jeon, S.-Y. Jang, *Energy Environ. Sci.* **2020**, *13*, 2915.

[6] H. Cheng, X. He, Z. Fan, J. Ouyang, *Adv. Energy Mater.* **2019**, *9*, 1901085.

[7] C. Cho, B. Kim, S. Park, E. Kim, *Energy Environ. Sci.* **2022**, *15*, 2049.

[8] X. He, H. Cheng, S. Yue, J. Ouyang, *J. Mater. Chem. A* **2020**, *8*, 10813.

[9] Q. Le, H. Cheng, J. Ouyang, *Chem. Eng. J.* **2023**, *469*, 143828.

[10] Q. Qian, H. Cheng, Q. Le, J. Ouyang, *Adv. Funct. Mater.*, *33*, 2303311.

[11] C.-G. Han, X. Qian, Q. Li, B. Deng, Y. Zhu, Z. Han, W. Zhang, W. Wang, S.-P. Feng, G. Chen, W. Liu, *Science* **2020**, *368*, 1091.

[12] Y. Li, Q. Li, X. Zhang, B. Deng, C. Han, W. Liu, *Adv. Energy Mater.* **2022**, *12*, 2103666.

[13] X. Lu, Z. Mo, Z. Liu, Y. Hu, C. Du, L. Liang, Z. Liu, G. Chen, *Angew. Chem. Int. Ed.* **2024**, *63*, e202405357.

[14] C.-G. Han, Y.-B. Zhu, L. Yang, J. Chen, S. Liu, H. Wang, Y. Ma, D. Han, L. Niu, *Energy Environ. Sci.* **2024**, *17*, 1559.

[15] Q. Le, H. Cheng, J. Ouyang, *DeCarbon* **2023**, *1*, 100003.

[16] Y. He, S. Li, R. Chen, X. Liu, G. O. Odunmbaku, W. Fang, X. Lin, Z. Ou, Q. Gou, J. Wang, N. A. N. Ouedraogo, J. Li, M. Li, C. Li, Y. Zheng, S. Chen, Y. Zhou, K. Sun, *Nano-Micro Lett.* **2023**, *15*, 101.
